# Supplementary material for: Competency-based assessment for the training of PhD students and early-career scientists
Source: eLife. 2018 May 31;7:e34801. doi: 10.7554/eLife.34801 (PMC6002247; doi:10.7554/eLife.34801)
Supplement: Table 1—source data 1. [file elife-34801-table1-data1.docx]

Supplementary Materials for

Competency-Based Assessment for PhD Scientists

M. F. Verderame, V. H. Freedman, L. M. Kozlowski, W. T. McCormack.

correspondence to: mxv8@psu.edu

**This PDF file includes:**

Table S1

Table S1.

Science PhD Core Competencies, Subcompetencies, and Milestones.

| **1. BROAD CONCEPTUAL KNOWLEDGE OF A SCIENTIFIC DISCIPLINE** | | | | | |
| --- | --- | --- | --- | --- | --- |
| **SUBCOMPETENCIES** | **MILESTONES** | | | | |
|  | **Beginning PhD Student** | **Advanced PhD Student** | **PhD**  **Graduate** | **Early Career Scientist** | **Science Professional** |
| **A. Knowledge base for multiple disciplines acquired from classes, seminars, journal clubs, etc.** | **Discuss**^1^ new knowledge | **Respond** to questions demonstrating understanding of new knowledge across disciplinary boundaries | **Formulate** questions that demonstrate understanding of new information and existing knowledge across disciplinary boundaries | **Articulate** connections between new information and existing knowledge across disciplinary boundaries | **Facilitate** discussions across disciplinary boundaries |
| **B. Broad scientific approaches** | **Understand** key basic principles and experimental bases for multiple disciplines | **Ask** relevant questions that relate multiple disciplines to research project | **Integrate** multiple disciplines into research | **Participate** in multi-disciplinary research | **Initiate and lead** multi-disciplinary research |

| **2. DEEP KNOWLEDGE OF A SPECIFIC FIELD** | | | | | |
| --- | --- | --- | --- | --- | --- |
| **SUBCOMPETENCIES** | **MILESTONES** | | | | |
|  | **Beginning PhD Student** | **Advanced PhD Student** | **PhD**  **Graduate** | **Early Career Scientist** | **Science Professional** |
| **A. Historical context of a specific area** | **Describe** major conceptual advances and progressive development of tools and approaches | **Incorporate** historical perspective and **acknowledge** prior contributions in scientific communications. | **Incorporate** historical perspective and **acknowledge** prior contributions in dissertation | **Educate** lab members and others about the historical context | **Review** oral and written scientific communications to ensure that background and significance reflects the historical context |
| **B. Current content expertise in the specific area** | **Perform** a literature search; **read, understand, and discuss** primary literature; **attend** seminars | **Recognize** important literature; **ask** seminar speakers relevant questions; **Discuss** key points; **Apply** to doctoral research | **Demonstrate** depth of knowledge by ability to **critically evaluate** papers, **question** dogma, **see** the big picture, **deliver** knowledgeable seminar | **Consistent use** of literature to inform research; quickly **acquire** new knowledge; **add** new information to seminar and collegial discussions | **Articulate** vision of research direction; **demonstrate** independent thinking and creativity; **teach and/or mentor** others |
| **C. Tools and approaches for a specific area** | **Use** existing experimental tools and approaches; **seek** help as needed | **Develop** new tools and/or approaches to investigate hypotheses  **Develop** a specific aim including hypothesis and experimental approaches | **Demonstrate** comprehensive knowledge of tools and approaches; **Assist** junior lab members in relevant techniques and approaches, both technically and theoretically | **Train** others in the use of relevant techniques and approaches | **Critically analyze** techniques, approaches, and data |

| **3. CRITICAL THINKING SKILLS** | | | | | |
| --- | --- | --- | --- | --- | --- |
| **SUBCOMPETENCIES** | **MILESTONES** | | | | |
|  | **Beginning PhD Student** | **Advanced PhD Student** | **PhD**  **Graduate** | **Early Career Scientist** | **Science Professional** |
| 1. **A. Recognize important questions** | **Describe** background information and **explain** rationale for an hypothesis | **Identify** important questions and hypotheses in a paper or experimental design | **Evaluate** results and **generate** new hypotheses based on historical and current context; **recognize** next important unanswered questions | **Consistently describe** experiments in terms of hypotheses and scientific method; **prioritize** research questions | **Independently generate** new hypotheses, **teach** the scientific method |
| **B. Design a single experiment (answer questions, controls, etc.)** | **Follow** experimental protocols, **seek** help as needed, **describe** critical role of controls | **Plan** experimental protocol; **include** relevant controls; **choose** appropriate methods; **troubleshoot** experimental problems | **Design and execute** hypothesis-based experiments independently; **evaluate** protocols of others; **imagine** range of experimental outcomes | Consistently **design and execute** experiments with appropriate controls; **assess** next steps; **critique** experiments of others | **Teach** experimental design; **guide** others doing experiments |
| **C. Interpret data** | **Describe** relationship between data and underlying experimental methods | **Understand** experimental methods, **evaluate** data for integrity and validity | Consistently **analyze and interpret** data; **recognize** significant results; **draw** appropriate conclusions | Independently **interpret** data; **use** data to inform experimental design | **Teach** others about data interpretation |
| **D. Design a research program** | **Participate** in discussions about research programs; **use** knowledge from literature to **ask** appropriate questions | **Recognize** connections and flow of experiments in a larger project; **explain** relationships among results | **Design** interrelated experiments to address an overarching question; **make** specific predictions and **define** alternative approaches based on results | **Plan** and **conduct** interrelated experiments needed to build a research program; **use** results to identify future research questions | Independently **design**, **plan** and **direct** a research program; **recognize** significant results and use to **build** future research programs |

| **4. EXPERIMENTAL SKILLS** | | | | | |
| --- | --- | --- | --- | --- | --- |
| **SUBCOMPETENCIES** | **MILESTONES** | | | | |
|  | **Beginning PhD Student** | **Advanced PhD Student** | **PhD**  **Graduate** | **Early Career Scientist** | **Science Professional** |
| 1. **A. Identify appropriate experimental protocols** | **Understand** how a specific experiment will answer a research question | **Design** experiments following established protocols; include appropriate positive and negative controls | **Explain** the advantages and disadvantages of several relevant protocols to address a specific question | Independently **choose** the most appropriate protocol | **Teach** the selection of appropriate experimental protocols |
| 1. **B. Design and execute experimental protocols** | **Observe** and independently **repeat** an existing experimental protocol, preparing necessary reagents | **Execute** an unfamiliar experimental protocol; **demonstrate** procedures; **assist** others with experimental protocols | **Modify** a familiar experimental protocol to meet current needs | **Build** a new protocol using parts of existing techniques to address a novel question | **Create** a novel protocol to answer a previously unanswerable question |
| 1. **C. Identify and troubleshoot technical issues** | **Replicate** experimental results; **recognize** when controls indicate technical problems | **Understand** underlying biochemical and technical aspects of protocols to **identify** sources of problems | **Execute** experiments properly based on troubleshooting experience | **Assist** others with troubleshooting techniques | **Teach** troubleshooting techniques |
| **D. Lab safety & regulatory issues** | **Complete required training** and **demonstrate** understanding of lab safety & regulatory policies | **Conduct** experiments in compliance with safe laboratory practices and policies | **Help** others follow safe lab practices; **assist** in writing relevant safety & regulatory protocols | **Write** relevant safety & regulatory protocols | **Teach** and **supervise** others about lab safety & regulatory policies |
| **E. Research records and data storage** | **Understand** critical nature of accurate record-keeping data security and access; **maintain** intact records of original data | **Understand** confidentiality issues in data; **increase awareness** of issues in research data keeping | **Teach** junior lab members the critical nature of accurate record-keeping data security and access | Supervise junior lab members on appropriate research record keeping; Be a role model | Develop appropriate protocols for storing data; teach accurate record-keeping; help colleagues address issues with data integrity |
| **F. Recognition of data ownership** | **Demonstrate understanding** of university policies and professional expectations on data ownership | **Understand** implications of intra- and inter-lab collaboration on data ownership & sharing in scientific and other communications | **Teach** junior lab members about data use and ownership | **Discuss** data ownership with collaborators; **respect** confidentiality and data ownership in peer review | **Proactively address** data ownership issues in an open and fair approach; **help** colleagues address issues with data ownership |

| **5. COMPUTATIONAL SKILLS** | | | | | |
| --- | --- | --- | --- | --- | --- |
| **SUBCOMPETENCIES** | **MILESTONES** | | | | |
|  | **Beginning PhD Student** | **Advanced PhD Student** | **PhD**  **Graduate** | **Early Career Scientist** | **Science Professional** |
| **A. Basic Statistical Analysis** | **Understand** different data types and how that informs test choice.  **Generate** and graph basic summary statistics from original data | **Select appropriate statistical test and design** experimental data collection to support ultimate statistical analysis **in consultation** with statistics expert to ensure proper choices. **Use** appropriate software tools to analyze data | **Independently select appropriate statistical test and design** experimental data collection to support ultimate statistical analysis.  **Recognize** when additional statistical consultation is necessary. | **Teach** trainees the value of statistical consultation | **Guide** trainees in choosing appropriate statistical tests and experimental designs in their research field. |
| **B. Bioinformatics Literacy** | **Understand** the basics tenets and paradigms of genome biology **including awareness of the complexity of information storage in biological systems.**  **Describe** the cross-disciplinary nature of bioinformatics.  **Locate** appropriate data repositories | **Recognize when** large-scale data-intensive biological problems are bioinformatics problems; seek expert support to determine appropriate analysis.  **Perform** basic data queries in public database. | **Use** appropriate databases, software tools, and algorithms relevant to research projects  **Identify** appropriate resources and experts to develop solutions to complex bioinformatics problems. | **Assist** beginning students in the conceptualization of bioinformatics problems. | **Assist** lab members and others in identifying appropriate bioinformatics resources and experts. |

| **6. COLLABORATION & TEAM SCIENCE** | | | | | |
| --- | --- | --- | --- | --- | --- |
| **SUBCOMPETENCIES** | **MILESTONES** | | | | |
|  | **Beginning PhD Student** | **Advanced PhD Student** | **PhD**  **Graduate** | **Early Career Scientist** | **Science Professional** |
| 1. **A. Openness to collaboration** | **Discuss** research effectively within own discipline | **Maintain** an open mind to clearly hear other perspectives; **recognize** that diverse opinions strengthens teams | **Seek help** across disciplines to solve research problems | **Demonstrate** broad intellectual curiosity to ask questions across disciplines | **Collaborate** effectively with colleagues from different disciplines |
| **B. Self-awareness** | **Recognize** personal strengths and weaknesses related to discipline-based research | **Recognize** personal strengths and weaknesses related to interdisciplinary research collaboration | **Understand** how own expertise can address a problem and how it differs from contributions of other disciplines | **Recognize** how the contributions of others can address a problem in interdisciplinary collaborations | **Subject** own disciplinary discovery to interpretation and scrutiny by researchers from other disciplines |
| **C. Disciplinary awareness** | **Demonstrate** general knowledge of own discipline | **Demonstrate** critical awareness of underlying assumptions of own discipline, its scope and contribution and limitations in addressing a given research question | **Share** research from own area of expertise in language meaningful to an interdisciplinary team | **Engage** colleagues from other disciplines to gain their perspectives on research problems, themes or topics | **Evaluate** the assumptions and limitations of other disciplines in interdisciplinary collaborative initiatives |
| **D. Integration** | **Develop** discipline-based research frameworks in collaboration with other scholars within own discipline | **Modify** own research as a result of interactions with colleagues from other fields | **Develop** interdisciplinary research frameworks in collaboration with scholars from other disciplines | **Integrate** concepts and methods from multiple disciplines in designing research protocols | **Collaborate** with others to integrate theories, methods and insights of multiple disciplines |
| **E. Team skills** | **Develop** personal team skills in order to strengthen team structure and dynamics | **Understand** strategies for interdisciplinary teamwork and communication including clarifying the meanings of key concepts and appreciating the perspectives of other disciplines | **Build** trust among collaborators in a diverse interdisciplinary team | **Contribute** to the creation of collective interdisciplinary knowledge that includes: thinking with team, adapting individual contributions, trusting value of other contributors, and negotiating differences | **Understand and effectively manage** conflict, feedback and credit relative to interdisciplinary team research |

| **7. RESPONSIBLE CONDUCT OF RESEARCH AND RESEARCH ETHICS** | | | | | |
| --- | --- | --- | --- | --- | --- |
| **SUBCOMPETENCIES** | **MILESTONES** | | | | |
|  | **Beginning PhD Student** | **Advanced PhD Student** | **PhD**  **Graduate** | **Early Career Scientist** | **Science Professional** |
| **A. Knowledge about responsible conduct of research (RCR)** | **Describe** rules and policies for ethical research practices | **Conduct** research following the rules and policies for ethical research | **Recognize** ethical and unethical research practices | **Teach** or lead discussions about rules and policies for ethical research practices | **Mentor** trainees in RCR |
| **B. Ethical decision making (EDM) in RCR**  **(outcome to process)** | **Identify** an ethical question (ethical sensibility: is there an ethical dilemma?); Know procedures for reporting and investigating research misconduct | **Know** one or more moral methods; Respond with correct answer to ethical issues; Understand that there might be multiple “right” answers | **Use** a moral method to address an ethical issue; Know that there are (might be) a plurality of views | **Recognize** ethical issues; Serve as resource or support for trainees confronting ethical issues | **Initiate** and/or lead discussions about how to confront ethical issues |
| **C. Moral Courage** | **Understand** that knowing what to do does not equal moral courage | **Seek** help for issues that confront you; **Respond** to hypothetical situations | **Report** unethical practices when encountered; **Recognize** that “authority figures” aren’t always “right” | **Be available** for junior trainees who are facing ethical challenges | **Share** your responses to prior ethical challenges with trainees |
| **D. Integrity** | **Know** the importance of character and being honest and fair | **Be conscious** of your own integrity | **Be conscious** of the integrity of those around you | **Be** a role model of integrity for junior trainees | **Be** a role model of integrity for trainees and peers |

| **8. COMMUNICATION SKILLS** | | | | | |
| --- | --- | --- | --- | --- | --- |
| **SUBCOMPETENCIES** | **MILESTONES** | | | | |
|  | **Beginning PhD Student** | **Advanced PhD Student** | **PhD**  **Graduate** | **Early Career Scientist** | **Science Professional** |
| **A. Informal Oral Presentation Skills** | **Introduce** journal article at journal club, including overview of figures; **present** results in laboratory (lab) meeting; **improve** English as a Second Language (ESL) skills (if applicable) | **Discuss** paper in-depth at journal club;  **prepare and practice** 2 minute elevator speech on own research to scientists or lay public; **discuss and evaluate** results at lab meeting; **answer** questions about own research spontaneously; **ask** relevant questions at a guest speaker seminar | **Interpret own data and relate** it to research in the field ; **critically analyze** other’s research and **fully participate** in lab meeting; **facilitate** thesis committee meeting; **lead** journal club; **apply knowledge to discuss** research with guest scientists and scientists at national meetings; **develop** job interview skills; **improve** ESL skills (if applicable) | **Work** with collaborators; **bring** broader scientific perspective to discussions; **participate in or lead** institutional committee meeting; **mentor** students and junior postdocs on informal presentation skills | **Mentor** junior scientists in developing speaking skills; **deliver** informal scientific talks with and without slides to various audiences; **lead** a group discussion; **participate** in discussions at national/international meetings |
| **B. Formal Oral Presentation Skills** | **Deliver** 15 minute oral presentation of thesis project (qualifying/ preliminary exam); **prepare** basic PowerPoint slides with narrative and data | **Present** research at works-in-progress seminar; **prepare** and present complex yet comprehensible ppt slides to describe research | **Present** research at full-length seminars; **deliver** platform presentation at national meetings; **mentor** junior students on presentation skills; **deliver** talk for a job interview, tailored to the audience | **Expertly present** own research in context of others’ work; **mentor** students and junior postdocs on formal presentation skills | **Mentor** junior scientists on formal presentation skills**; critique** presentations by lab members and colleagues; **develop** experience and proficiency in communication of your science and the “big picture.” |
| **C. Written Communication - Scientific Manuscript** | **Learn** guidelines regarding use of material from publications, including what plagiarism is; **learn** how to properly cite references; **learn** how to abstract material from the literature; **learn** the structure of a scientific publication | **Outline** own research for manuscript; **learn** how to prepare manuscript for particular journal; **analyze** own data and **prepare** figures for manuscript; **learn** how to write introduction and discussion | **Write** literature review and **prepare** manuscript(s) for publication with mentor’s help; **draft** response to editors’ queries and critiques of submitted manuscripts; **write** thesis, following style guidelines; **show** junior students how to prepare manuscript | **Integrate** own research with broader general scientific context; **participate** in review of laboratory manuscripts; **mentor** students and junior postdocs on manuscript writing; **learn** how to review manuscripts | **Review** manuscripts for publication, including writing constructive critiques; **respond** to critiques appropriately; **mentor** lab members on manuscript writing and review |
| **D. Written Communication - Grant Proposals** | **Learn** structure of fellowship proposal; **learn how to write** specific aims; **write** a preliminary/qualifying exam proposal in fellowship or grant format | **Search** for funding opportunities; **learn how to read** a Request For Applications; **write** fellowship application with mentor | **Write** fellowship application independently for review with mentor; **mentor** junior students on finding funding opportunities and grant writing | **Learn how to review** grants; **critique** fellowship applications and grants for others in lab; **develop** specific aims for future independent research | **Review** grants; **sit** on study sections; **communicate** constructive criticisms; respond to critiques appropriately; **mentor** lab members on grant writing and review |
| **E. Written Communication – Meeting Poster** | **Distinguish** between slide presentation and poster presentation; **attend** local poster sessions; **participate** in poster preparation by senior lab members | **Prepare** meeting poster with mentor’s help; **develop** proficiency at discussing results during poster session | **Prepare** meeting poster independently; **discuss** results at poster session; **mentor** junior students on poster preparation | **Design** captivating meeting poster; **listen and evaluate** poster presentation of junior lab members | **Mentor** lab members on poster presentation; **critique** poster; **listen and critique** practice poster presentation |
| **F. Communication with the Public** | **Begin to think about** important topics in field of interest and how to present these topics to scientists in other fields and non-scientists | **Design and practice** 2-minute elevator speech to explain research to scientists in other fields and to non-scientists | **Communicate** short and long descriptions of science to variety of lay audiences; **explain** broader context of own research; **participate** in outreach activities to local schools and community groups; | **Lead** outreach activities to local schools, community groups; **mentor** junior lab members on presentation preparation; **participate** in national outreach programs; **learn how to interact** with media professionals | **Interact** with institutional and external media professionals to describe research; **participate** in local and national outreach activities; **provide** scientific expertise as speaker |

| **9. Leadership Skills** | | | | | |
| --- | --- | --- | --- | --- | --- |
| **SUBCOMPETENCIES** | **MILESTONES** | | | | |
|  | **Beginning PhD Student** | **Advanced PhD Student** | **PhD**  **Graduate** | **Early Career Scientist** | **Science Professional** |
| **A. Vision** | **Articulate** the lab's research direction(s) and their possible role(s) within it | **Explain** own research agenda and role in larger research picture; **beginning to develop** a vision for future career path | **Articulate** future directions of research project and goals for career direction | **Help** junior lab members understand lab’s research agenda; **apply** for positions based on research goals and desired career path | **Articulate** overall direction and clear goals to research team and to others via presentations and proposals |
| **B. Integrity** | **Learn** institutional and cultural norms of the research community | **Consistently adhere** to rules, regulations, and ethical principles of research conduct; **Is** dependable and trustworthy; **Is** accountable for own actions | **Teach** junior lab members rules, regulations, and ethical principles of research conduct | **Hold** others accountable for their actions | **Model** dependability and trustworthiness; mentors lab members and others on institutional and cultural norms of research community |
| **C. Group dynamics and interpersonal skills** | **Contribute** to group/team discussion when prompted; **Learn** about interpersonal relations within the lab and/or institution including cultural diversity | **Contribute** freely to group/team discussions; **Is** respectful of others’ ideas; **Is** well prepared for committee meetings | **Coordinate** group efforts to achieve goals given clear guidelines; **Lead** others toward achieving goals; **Assign** tasks by seeking volunteers, delegating as needed | **Listen** actively and **show understanding** by acknowledging and building on others’ ideas; **Encourage** others’ participation; **Give** recognition and encouragement | **Involve** group in setting goals; **Organize** group for planning to achieve goals; **Lead** others to work together; Intervenes when tasks are not moving toward goals |
| **D. Organization and planning** | **Prioritize & coordinate** own tasks within the overall lab program under supervision | **Prioritize & coordinate** own tasks, **integrate** contributions into the overall program with minimal supervision; **effectively manage** time | **Prioritize & coordinate** own tasks, **integrate** contributions into the overall program independently; **Assist** junior lab members with organization and planning skills | **Assist** junior lab members with time and project management | **Prioritize & coordinate** overall research program; **Mentor** junior lab members with time and project management |
| **E. Decision-making** | **Voice** opinion when prompted | **Voice** opinion without prompting; **explore** multiple perspectives and **seek** feedback about possible decisions | **Make** responsible and good decisions;  **demonstrate** confidence by defending decisions | **Assist** junior lab members with learning decision making process | **Mentor** lab members on decision making skills; **Provide** praise when good decisions are made |
| **F. Problem-solving** | **Contribute** information, **brainstorm** solutions, and **assist** in evaluating alternatives when prompted | **Contribute** information, **brainstorm** solutions, **evaluate** alternatives independently; **Build** on others’ ideas; **Articulate** problem-solving process | **Offer** insightful or creative solutions, and **provide** a framework for evaluating alternatives; **assist** junior lab members with problem solving process | **Solve** own problems with minimal assistance; **support** lab members with problem-solving process | **Lead and educate** research team on problem-solving process; **evaluate** and **select** most effective approach to solve problems; **collaborate** with others on problem-solving |
| **G. Managing Conflicts** | **Learn** to work effectively with others: **recognize** conflicts, **seek** advice in difficult situations to avoid escalation | **Learn** to mediate conflict situations by emphasizing goals / issues rather than personalities; **Offer** solutions; **Maintain** collegiality | **Participate in** conflict resolution and **guide** others to a collegial research environment | **Resolve** conflict based upon objective criteria and varied approaches | **Mentor** others in difficult situations and conflict resolution |

| **10. Survival Skills** | | | | | |
| --- | --- | --- | --- | --- | --- |
| **SUBCOMPETENCIES** | **MILESTONES** | | | | |
|  | **Beginning PhD Student** | **Advanced PhD Student** | **PhD**  **Graduate** | **Early Career Scientist** | **Science Professional** |
| **A. Motivation** | **Accept direction** passively; **attend** required scientific functions when prompted | **Attend** required scientific functions without being prompted; **Display curiosity** about science via discussion | Self-driven for planning and direction; **attend** required and elective scientific functions of value to training; **display passion** for science; **share excitement** for science with others | Actions reflect goals beyond what is required; **proactive** in problem-solving and decision-making; **influence** events to reach goals | Spontaneously take advantage of opportunities; **role model** for self-motivation, and able to **mentor** others |
| **B. Perseverance** | **Complete first-year curriculum** in the face of the unique pressures and demands of graduate school and research | Willingly **repeat experiments** as needed to find answers; begin to **develop** alternative strategies to overcome obstacles | **Complete** dissertation research in the face of continued pressures and demands; **Engage** others for support; **assist** junior lab members with coping mechanisms for pressures and demands | **Educate** junior lab members on the pressures and demands and coping mechanisms; play a junior **mentoring** role | **Mentor** others on coping mechanisms for pressures and demands; continue active career in biomedical science |
| **C. Adaptability** | **Adapt** to graduate-level coursework; **Manage** complex schedule of classes, rotation research, and other time demands | **Change priorities** to meet varying demands; **adjust approach** to match varied task requirements | **Adjust own behavior** to work with others and adapt to new situation | **Adjust** quickly to new responsibilities and tasks; **assist** junior lab members to adapt to new situations | **Adapt** effectively to varying responsibilities & tasks; **mentor** others to adapt to new situations |
| **D. Professional Development** | **Attend** required professional and/or career development courses or seminars; Becoming **self-aware** of development as a scientist | **Use** Individual Development Plan (IDP) appropriate to stage of training | Has **awareness** of research organization and roles within it; **seek** additional opportunities for professional development relevant to desired career pathways | **Aware** of own professional development needs for desired career pathway; **use** Individual Development Plan (IDP) appropriate to stage of training | **Seek** continuing professional development opportunities for self-improvement; **mentor** others about professional and career development |
| **E. Networking** | **Get to know** peers.  **Meet** faculty. | **Get to know** members of your thesis committee and other faculty | **Talk** with scientists at national meetings about both your research and their research | **Identify and communicate** with potential future colleagues / co-workers. | **Identify and develop** relationships with collaborators. |

^1^Verbs in bold font indicate observable behaviors representing each stage of skill acquisition.
